# Changes in the health systems and policy environment for maternal and newborn health, 2008–2018: An analysis of data from 78 low-income and middle-income countries

**DOI:** 10.1016/j.socscimed.2023.115765

**Published:** 2023-03

**Authors:** Elizabeth K. Stierman, Blerta Maliqi, Meighan Mary, Martin AJ. Dohlsten, Elizabeth Katwan, Allisyn C. Moran, Andreea A. Creanga

**Affiliations:** a Department of International Health, Johns Hopkins Bloomberg School of Public Health, 615 N. Wolfe Street, Baltimore, MD 21205, USA; b International Center for Maternal and Newborn Health, Johns Hopkins Bloomberg School of Public Health, 615 N Wolfe Street, Baltimore, MD 21205, USA; c Department of Maternal, Newborn, Child, Adolescent Health and Ageing, World Health Organization, Avenue Appia 20, 1211 Geneva, Switzerland; d Department of Gynecology and Obstetrics, Johns Hopkins School of Medicine, 733 N. Broadway, Baltimore, MD 21205, USA

**Keywords:** Maternal health, Newborn health, Health policy, Health systems, LMICs

## Abstract

**Background:**

Political, social, economic, and health system determinants play an important role in creating an enabling environment for maternal and newborn health. This study assesses changes in health systems and policy indicators for maternal and newborn health across 78 low- and middle-income countries (LMICs) during 2008–2018, and examines contextual factors associated with policy adoption and systems changes.

**Methods:**

We compiled historical data from WHO, ILO, and UNICEF surveys and databases to track changes in ten maternal and newborn health systems and policy indicators prioritized for tracking by global partnerships. Logistic regression was used to examine the odds of systems and policy change based on indicators of economic growth, gender equality, and country governance with available data from 2008 to 2018.

**Results:**

From 2008 to 2018, many LMICs (44/76; 57·9%) substantially strengthened systems and policies for maternal and newborn health. The most frequently adopted policies were national guidelines for kangaroo mother care, national guidelines for use of antenatal corticosteroids, national policies for maternal death notification and review, and the introduction of priority medicines in Essential Medicines Lists. The odds of policy adoption and systems investments were significantly greater in countries that experienced economic growth, had strong female labor participation, and had strong country governance (all p < 0·05).

**Conclusions:**

The widespread adoption of priority policies over the past decade is a notable step in creating an environment supportive for maternal and newborn health, but continued leadership and resources are needed to ensure robust implementation that translates into improved health outcomes.

## Highlights

• From 2008 to 2018, many LMICs adopted policies supportive of maternal and newborn health.
• Many adopted guidelines for kangaroo mother care and use of antenatal corticosteroids.
• Many also adopted national policies for maternal death notification and review.
• The odds of policy adoption were greater in certain contexts.
• Economic growth, gender equality, and governance may facilitate policy adoption.

## 1 Introduction

The world has seen substantial progress in maternal and newborn survival in recent decades. The global maternal mortality ratio declined by 38 percent between 2000 and 2017 (World Health Organization, 2019), and neonatal mortality rates dropped 43 percent between 2000 and 2020 (UNICEF, WHO, World Bank Group, United Nations, 2020). However, progress is uneven, and many countries will not reach the 2030 Sustainable Development Goal (SDG) targets for maternal and neonatal mortality reductions if current trends continue (UNICEF, WHO, World Bank Group, United Nations, 2020; World Health Organization, 2021a).

A multitude of diverse factors contribute to the success or failure of countries to achieve maternal and newborn survival goals (Kuruvilla et al., 2014; Bishai et al., 2016). These include proximal factors, such as the coverage and quality of key maternal and newborn health (MNH) interventions, and distal factors, such as political, social, economic, and health system determinants (Moran et al., 2016; Jolivet et al., 2018; Singh et al., 2016). In the past, global strategies tended to emphasize actions to improve coverage of clinical interventions to address the proximal causes of maternal and neonatal mortality, but there is increasing recognition that action on distal determinants is also required to achieve the SDGs. This emerging consensus is evident in the milestones adopted by global multi-partner initiatives—Strategies for Ending Preventable Maternal Mortality (EPMM) and Every Newborn Action Plan (ENAP)—which emphasize the broader elements of policy and health systems investments required to optimize MNH (World Health Organization, 2017; World Health Organization & UNFPA, 2021). These initiatives seek to accelerate progress towards the targets set forth in the SDGs and the Global Strategy for Women's, Children's and Adolescents' Health (“Global Strategy”).

**Fig. 1:**
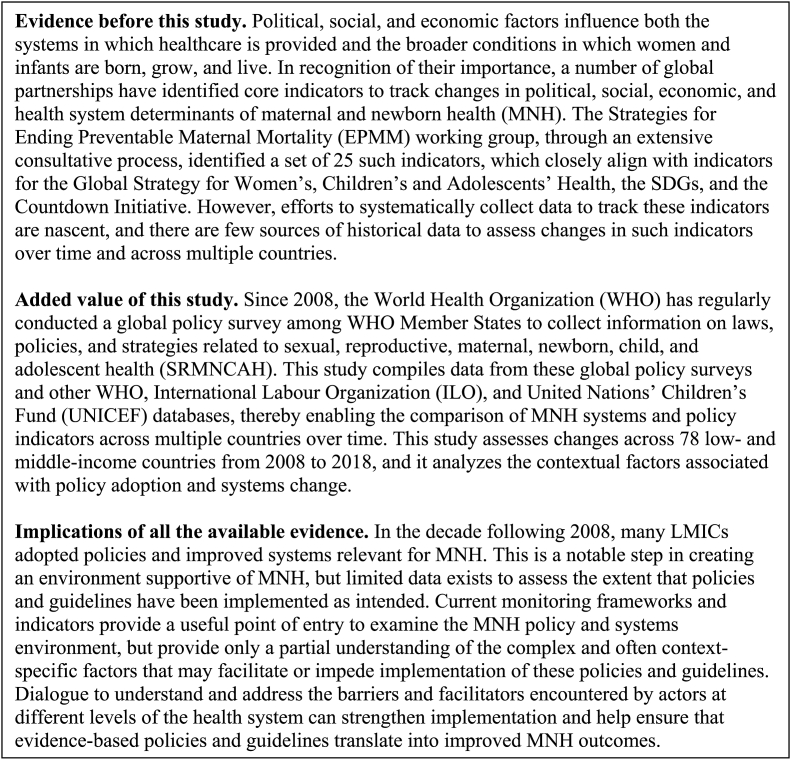
Research in context.

The increasing recognition of the importance of the health systems and policy environment has fueled demand for data to monitor policy adoption and health systems investments, but until recently, limited data were available for multi-country comparisons or for tracking trends over time (Fig. 1). This study compiles historical data on MNH systems and policy indicators from the World Health Organization's (WHO) Sexual, Reproductive, Maternal, Newborn, Child, and Adolescent Health (SRMNCAH) policy surveys and other WHO, International Labour Organization (ILO), and United Nations' Children's Fund (UNICEF) databases. Our aims are to assess changes in MNH systems and policy indicators across 78 low- and middle-income countries (LMICs) during 2008–2018, and to understand the contextual factors associated with policy adoption and systems change.

## 2 Methods

We used country indicators from three rounds of WHO SRMNCAH policy surveys and other WHO, ILO, and UNICEF databases for corresponding years. All WHO member states were eligible to participate in SRMNCAH policy surveys (World Health Organization, 2020a). The analytical sample for this study was restricted to LMICs who completed the 2018 SRMNCAH policy survey and also had historical information available from 2008 to 2014-2014/2015 survey rounds. Country income groupings used throughout the analyses were based World Bank categories for 2018 (World Bank Group, 2018).

### 2.1 Selection of indicators

To assess changes in MNH systems and policies, we compiled data on ten tracer indicators (Supplementary Table S1). These represent a subset of existing indicators prioritized for global tracking by the EPMM, ENAP, and the Countdown Initiative. The selection of indicators was not intended to capture all policies and systems that may impact MNH; rather, selection aimed to capture priority indicators representing each of the WHO health systems building blocks and for which data were available for the reference time period. Of these ten indicators, two were categorical variables measuring the extent national legislation was aligned with international conventions for (a) paid maternity leave and (b) marketing of breastmilk substitutes. Six were binary variables indicating the existence (or not) of (c) national guidelines on kangaroo mother care for clinically stable low birthweight newborns, (d) national guidelines on use of antenatal corticosteroids for preterm labor, (e) priority maternal medicines included on Essential Medicines Lists (EMLs), (f) priority newborn medicines and commodities included on EMLs (or national commodity lists), (g) national policies on maternal death notification, and (h) national policies on maternal death reviews. The last two were continuous variables indicating (i) health worker density per 10,000 population and (j) domestic general government health expenditures (GGHE) per capita.

We also identified contextual factors related to economic growth, gender equality, and country governance, hypothesized to influence adoption of MNH policies and health system investments. These included gross domestic production (GDP) growth per capita, percentage of seats held by women in national parliaments, labor force participation by females, and composite governance scores for voice and accountability, political stability and absence of violence, government effectiveness, regulatory quality, rule of law, and control of corruption.

### 2.2 Data sources and data validation procedures

Data sources included WHO SRMNCAH policy surveys, WHO Global Health Workforce Statistics Database, WHO Global Health Expenditure Database (GHED), WHO Global Maternal Death Surveillance and Response (MDSR) implementation survey, WHO & UNICEF Marketing of Breast-milk Substitutes: National Implementation of the International Code Status Reports, ILO World Social Protection Reports, ILO Working Conditions Laws Database, and the ILO NORMLEX database (Supplementary Table S1). (World Health Organization, 2020a; World Health Organization, 2016d; World Health Organization, 2021b; World Health Organization, 2021c; World Health Organization, 2016c; World Health Organization, 2020b; World Health Organization, 2018; World Health Organization, 2016a; World Health Organization, 2013a; International Labour Organization, 2021; International Labour Organization, 2017; International Labour Organization, 2014; International Labour Organization 2; International Labour Organization, 2022) Additional information on early adopters of national guidelines for kangaroo mother care and antenatal corticosteroids was found through the Healthy Newborn Network (Maternal and Child Health Integrated Program MCHIP, 2012; Healthy Newborn Network, 2013; McCord et al., 2016; Healthy Newborn Network, 2012). In addition, data on economic growth, gender equality, and country governance were sourced from the World Bank's World Development Indicators database and Worldwide Governance Indicators project (World Bank; Worldwide Governance Indicators, 2021).

After compiling data on selected indicators for all available years between 2008 and 2018, we reviewed changes in each indicator across years and across data sources on a country-by-country basis. We flagged any inconsistencies that might indicate a reporting error (e.g., a country reporting a national policy for review of maternal deaths in the 2015 MDSR survey but reporting there was no such policy in the 2018 SRMNCAH survey). If an inconsistency was found, we reviewed the relevant source documents (e.g., national policy, guidelines) issued by the country to validate the data; if warranted, we recoded variables to align with the content and publication date of the original source document(s).

### 2.3 Statistical analyses

We described each indicator for available years between 2008 and 2018. Data for indicators collected from surveys were generally available at three time points: 2008, 2014/15, and 2018; countries that did not respond to surveys in a given year were reported as missing and excluded from the calculation of percentages for the corresponding year. Other indicators were generally available annually. To smooth fluctuations and provide estimates for missing years, we fit mixed-effects models to, first, model the average change in health workforce density over time for a given country, and then separately, model the average change in GGHE per capita over time for a given country. This approach to modeling multilevel, longitudinal data allows us to represent country differences with a random intercept and, also, to model country-specific trends over time with a random slope (i.e., random coefficient). (Rabe-Hesketh and Skrondal, 2012).

Next, we classified how each indicator changed over the time period on a country-by-country basis, comparing data for the earliest available year (usually 2008) to 2018 data. For binary indicators measuring policy adoption, we categorized changes as: (a) already in existence since 2008 (or the earliest available year); (b) adopted by 2018; or (c) not adopted by 2018. For categorical indicators measuring alignment of legislation with international conventions, we categorized as: (a) legislation already ratified or substantially aligned in 2008; (b) strengthened during 2008–2018; or (c) not strengthened during 2008–2018. Changes in health worker density were categorized in the following manner: (a) already attained SDG index threshold of at least 44.5 doctors, nurses, and midwives per 10,000 population in 2008 and remained at or above threshold; (b) above average increase in health worker density during 2008–2018 relative to other countries in the sample; (c) minimal to average increase during 2008–2018; or (d) decrease during 2008–2018. Similarly, we categorized changes in GGHE per capita as: (a) country invested at least $150 per capita in 2008 and value remained at threshold or increased; (b) above average growth in GGHE per capita during 2008–2018 relative to other countries in the sample; (c) minimal to average growth during 2008–2018; or (d) decrease during 2008–2018.

We then summarized the data to classify countries into five categories based on the overall level of change in the MNH systems and policy environment: “remained strong” (i.e., at least six of ten tracer indicators were already in existence or had attained the target threshold in 2008); “strengthened” (i.e., at least four indicators adopted/strengthened during 2008–2018); “limited change” (i.e., three or fewer indicators adopted/strengthened during 2008–2018); “reversals” (i.e., at least one indicator with a substantial reversal and few indicators adopted/strengthened); or “mixed changes” (i.e., at least one indicator with a substantial reversal and several indicators adopted/strengthened) (see Supplementary Table S2 for full definitions). Two countries (Lesotho and Democratic People's Republic of Korea) were not given an overall classification due to lack of data or challenges in validating data for multiple indicators.

Logistic regression was used to model the odds of substantial MNH systems and policy change based on indicators of economic growth, gender equality, and country governance. In our modeling, countries were considered to experience the outcome if they had substantially “strengthened” MNH systems and policies during 2008–2018 (n = 44). The reference group included countries that had “limited change”, “mixed changes”, or “reversals” (n = 30), with a total sample size of 74 countries. Countries with a strong MNH systems and policy environment in 2008 that “remained strong” were excluded from the model, because they were considered as having reached a ceiling wherein further policy adoption and systems investments would no longer be feasible, irrespective of continued improvements in economic growth, gender equality, or governance.

We hypothesized that having high levels of economic growth, gender equality, and country governance in 2008 may increase the likelihood of MNH systems strengthening and policy adoption. We also hypothesized that changes in indicators of economic growth, gender equality, and country governance during 2008–2018 (e.g., a large increase in female parliamentary representation) may affect the likelihood of MNH systems and policy change. We fit multiple univariable regression models to estimate the odds of substantial MNH systems and policy change based on the initial 2008 value of each indicator and, separately, the change in each indicator's value between 2008 and 2018. Changes in GDP per capita were measured as the difference in natural log (ln) GDP per capita between 2008 and 2018, rescaled to measure incremental changes of 0.1 ln-units, which is equivalent to a change of 10.5% in GDP per capita. Changes in female parliamentary representation were measured as the absolute difference in percentages between 2008 and 2018. Governance scores and female labor participation were relatively stable during 2008–2018, so we did not assess whether changes in these indicators were associated with the outcome (Supplementary Table S3).

We used Stata version 15.1 for all statistical analyses (Rabe-Hesketh and Skrondal, 2012). The 2018–2019 WHO SRMNCAH policy survey followed necessary WHO protocols for non-emergency, non-human-subject data collection.

### 2.4 Sensitivity analysis

Sensitivity analysis applied an alternative approach to examine whether MNH systems and policies were associated with indicators of economic growth, gender equality, and country governance. For regression modeling, we specified the outcome as the number of MNH systems and policies in existence—or which had reached a minimum threshold—at the end of the reference period in 2018, with a maximum score of 10 indicating the country had all ten MNH systems and policy tracer indicators. A linear regression model examined the association between having a greater number of MNH systems and policies in existence and indicators of economic growth, gender equality, and country governance.

### 2.5 Role of funding source

The funder of the study had no role in study design, data collection, data analysis, data interpretation, or writing of the report.

## 3 Results

Of the 136 WHO member countries classified as LMICs, 112 (82%) responded to the 2018 SRMNCAH survey and 78 (58%) also had historical information available on MNH systems and policy indicators. Our analysis focuses on these 78 countries (Table 1). Compared to the larger pool of 136 WHO member countries classified as LMICs, the regional and income distribution of our 78-country sample was generally similar, but had relatively higher representation from the WHO Africa region and lower representation from the WHO Americas region; it also had a higher proportion of lower-income countries.

**Table 1:** Sample characteristics.

	Countries (n = 78)
	n (%)
WHO REGION	
Africa	38 (48.7)
Americas	7 (9.0)
Eastern Mediterranean	11 (14.1)
Europe	6 (7.7)
South Eastern Asia	9 (11.5)
Western Pacific	7 (9.0)
**INCOME CLASSIFICATION**^a^	
Low income	29 (37.2)
Lower middle-income	34 (43.6)
Upper middle-income	15 (19.2)

a Income classification as listed by the World Bank for 2018.

Between 2008 and 2018, many countries adopted key MNH policies and invested in systems relevant for MNH (Table 2; see also Supplementary Table S4). The most frequently adopted policies were national guidelines for kangaroo mother care, national guidelines for use of antenatal corticosteroids, national policies for maternal death notification and review, and the inclusion of priority MNH medicines in EMLs. Few countries had national guidelines on kangaroo mother care (n = 2; 2·6%) or the use of antenatal corticosteroids (n = 1; 1·3%) in 2008, but nearly all countries had adopted these guidelines by 2018 (64/76 or 84·2% and 69/76 or 90·8%, respectively). Likewise, while approximately one-third of countries had national policies for maternal death notification and review in 2008, nearly all had such policies by 2018 (75/78 or 96·2% and 76/78 or 97·4% respectively). In 2015, the majority (42/69; 60·9%) of countries had all three priority maternal medicines (i.e., oxytocin, misoprostol, and magnesium sulphate) included in EMLs, but fewer (5/68; 7·4%) had priority newborn medicines (i.e., injectable antibiotics, antenatal corticosteroids, chlorhexidine, and resuscitation equipment). By 2018, most countries (57/72; 79·2%) included all four newborn medicines and commodities in EMLs and national lists of commodities; among those that did not, chlorhexidine and neonatal resuscitation equipment were the items less frequently available.

**Table 2:** MNH systems and policy indicators, 2008–2018.

	2008 (n = 77)	2014/15^a^ (n = 78)	2018 (n = 78)
LEGISLATIVE CONTEXT	n (%)	n (%)	n (%)
Paid maternity leave legislation in alignment with ILO Convention 183 (C183)			
Ratified ILO C183	2/76 (2.6)	6/77 (7.8)	8/77 (10.4)
Substantially aligned^b^	15/76 (19.7)	14/77 (18.2)	17/77 (22.1)
Moderately aligned^c^	22/76 (29.0)	19/77 (24.7)	21/77 (27.3)
Few or no provisions in law^d^	37/76 (48.7)	38/77 (49.3)	31/77 (40.3)
Legal status of International Code of Marketing of Breast-milk Substitutes^e^			
Substantially aligned with the Code	11 (14.3)	16 (20.5)	17 (21.8)
Moderately aligned with the Code	18 (23.4)	25 (32.1)	25 (32.1)
Some provisions of Code adopted	15 (19.5)	15 (19.2)	15 (19.2)
No provisions in law	33 (42.9)	22 (28.2)	21 (26.9)
**MNH GOVERNANCE AND LEADERSHIP**			
National guidelines recommend Kangaroo Mother Care for clinically stable low birthweight newborns	2 (2.6)	40/65 (61.5)	64/76 (84.2)
National guidelines recommend use of antenatal corticosteroids for preterm labor	1 (1.3)	39/55 (70.9)	69/76 (90.8)
**MNH MEDICINES AND COMMODITIES**			
Priority maternal medicines included in the EML^f^	NA	42/69 (60.9)	73 (93.6)
Priority newborn medicines included in the EML^g^	NA	5/68 (7.4)	57/72 (79.2)
**MATERNAL DEATH SURVEILLANCE AND RESPONSE**			
National policy to notify all maternal deaths^h^	27/68 (39.7)	69 (88.5)	75 (96.2)
National policy to review maternal deaths	17/52 (32.7)	49/55 (89.1)	76 (97.4)
**HEALTH WORKFORCE**	**Median (IQR)**	**Median (IQR)**	**Median (IQR)**
Aggregate density of physicians, nurses and midwives per 10,000 population^i^	11.9 (6.1–29.1)	15.1 (7.7–30.8)	17.0 (9.1–32.3)
**HEALTH FINANCING**			
Domestic general government health expenditures, per capita in PPP international dollars^i^	41.3 (21.5–155.6)	60.7 (24.0–189.5)	71.2 (27.8–214.7)

Acronyms: EML, Essential Medicine List. ILO, International Labour Organization. IQR, interquartile range. MNH, maternal and newborn health. NA, not applicable. PPP, purchasing power parity.

Notes: Sample includes 77 countries prior to 2011, and 78 countries in 2011 or later years; South Sudan became an independent country in 2011.

a 2014 values shown for all indicators except national guidelines (kangaroo mother care, antenatal corticosteroids), priority medicines (maternal, newborn), and national policies (maternal death notification, maternal death review), which reflect 2015 value.

b Country passed national legislation that is aligned with three key provisions of ILO convention 183 (14 weeks of maternity leave, paid at 66% of previous earnings or higher, fully financed by social insurance or public funds).

c Country passed national legislation that is aligned with three key provisions of the earlier ILO Convention 103 (12 weeks of maternity leave, paid at 66% of previous earnings or higher, at least partially financed social insurance or public funds).

d Country had not passed national legislation on maternity leave, or country passed legislation that only weakly aligns with ILO conventions (e.g., program is employer liability).

e Classification of legislation harmonized with definitions used in the Marketing of Breast-Milk Substitutes: National Implementation of the International Code Status Report, 2020 (WHO, 2020b); previous years were updated to match 2020 definitions.

f Country had all 3 of the following in the Essential Medicines List: oxytocin, misoprostol, and magnesium sulphate.

g Country had all 4 of the following in the Essential Medicines List (or National Commodities List): injectable antibiotics, antenatal corticosteroids, chlorhexidine, resuscitation equipment.

h Prior to 2018, the survey asked whether the country has a national policy to notify all maternal deaths. In 2018, the wording changed to ask whether the country has a national policy requiring maternal deaths be notified within 24 h to a central authority. To match historical data, we verified and then recoded 4 entries in the 2018 data to indicate whether the country has any policy on maternal death notification without requiring a specific timeframe within 24 h.

i Annual country estimates predicted using a mixed-effects model with a random intercept for country and random slope for time.

Fewer changes were seen in the legislative context relative to other policy areas. Only one-fifth (16/77; 20·8%) of countries strengthened legal provisions for paid maternity leave (Table 3). Some progress was seen in the adoption of legislation aligned with the International Code of Marketing of Breastmilk Substitutes, but change was likewise limited; only one-quarter (18/78; 23·1%) of countries strengthened such provisions. Mixed changes were seen in health workforce density and government health spending per capita. Several countries (12/72; 16·7%) maintained the SDG threshold of at least 44.5 doctors, nurses, and midwives per 10,000 population throughout the timeframe, but a similar share (10/72; 13·9%) saw decreases in their health workforce per capita (Table 3). Similarly, while most countries experienced growth in GGHE per capita, some countries (14/72; 19·4%) saw declining expenditures in real terms. Decreases in government health expenditures were more common in low-income countries relative to upper middle-income countries, as classified by the World Bank income groupings.

**Table 3:** Changes in MNH systems and policy indicators by income group, 2008–2018.

	Low income (n = 29)	Lower middle income (n = 34)	Upper middle income (n = 15)	All countries (n = 78)
	n (%)	n (%)	n (%)	n (%)
LEGISLATIVE CONTEXT				
Paid maternity leave legislation-ILO C183				
Ratified in or prior to 2008	1 (3.5)	1 (2.9)	0/14 (0.0)	2/77 (2.6)
Strengthened during 2008–2018	5 (17.2)	7 (20.6)	4/14 (28.6)	16/77 (20.8)
Not strengthened during 2008–2018	23 (79.3)	26 (76.5)	10/14 (71.4)	59/77 (76.6)
Legal status of International Code of Marketing of Breastmilk Substitutes				
Substantially aligned in 2008	3 (10.3)	5 (14.7)	3 (20.0)	11 (14.1)
Strengthened during 2008–2018	5 (17.2)	9 (26.5)	4 (26.7)	18 (23.1)
Not strengthened during 2008–2018	21 (72.4)	20 (58.8)	8 (53.3)	49 (62.8)
**MNH GOVERNANCE AND LEADERSHIP**				
National guidelines recommend Kangaroo Mother Care for clinically stable low birthweight newborns				
Guidelines in existence since 2008	1/28 (3.6)	0 (0.0)	1/14 (7.1)	2/76 (2.6)
Guidelines adopted during 2008–2018	23/28 (82.1)	28 (82.4)	11/14 (78.6)	62/76 (81.6)
Guidelines not adopted by 2018	4/28 (14.3)	6 (17.7)	2/14 (14.3)	12/76 (15.8)
National guidelines recommend use of antenatal corticosteroids for preterm labor				
Guidelines in existence since 2008	0/28 (0.0)	0/33 (0.0)	1 (6.7)	1/76 (1.3)
Guidelines adopted during 2008–2018	25/28 (89.3)	30/33 (90.9)	13 (86.7)	68/76 (89.5)
Guidelines not adopted by 2018	3/28 (10.7)	3/33 (9.1)	1 (6.7)	7/76 (9.2)
**MNH MEDICINES AND COMMODITIES**				
Priority maternal medicines included in the EML^a^				
All included in EML since 2014	17 (58.6)	19 (55.9)	6 (40.0)	42 (53.9)
Added to EML during 2014–2018	11 (37.9)	12 (35.3)	8 (53.3)	31 (39.7)
Not all included in EML in 2018	1 (3.5)	3 (8.8)	1 (6.7)	5 (6.4)
Priority newborn medicines included in the EML^b^				
All included in EML since 2014	3/26 (11.5)	2/32 (6.3)	0/14 (0.0)	5/72 (6.9)
Added to EML during 2014–2018	18/26 (69.2)	24/32 (75.0)	10/14 (71.4)	52/72 (72.2)
Not all included in EML in 2018	5/26 (19.2)	6/32 (18.8)	4/14 (28.6)	15/72 (20.8)
**MATERNAL DEATH SURVEILLANCE AND RESPONSE**				
National policy to notify all maternal deaths^c^				
Policy in existence since 2008	7 (24.1)	16 (47.1)	11 (73.3)	34 (43.6)
Policy adopted during 2008–2018	20 (69.0)	17 (50.0)	4 (26.7)	41 (52.6)
Policy not adopted by 2018	2 (6.9)	1 (2.9)	0 (0.0)	3 (3.9)
National policy to review maternal deaths				
Policy in existence since 2008	9 (31.0)	16 (47.1)	11 (73.3)	36 (46.2)
Policy adopted during 2008–2018	19 (65.5)	18 (52.9)	3 (20.0)	40 (51.3)
Policy not adopted by 2018	1 (3.5)	0 (0.0)	1 (6.7)	2 (2.6)
**HEALTH WORKFORCE**				
Aggregate density of physicians, nurses and midwives per 10,000 population				
SDG threshold met and maintained since 2008^d^	2/26 (7.7)	5/32 (15.6)	5/14 (35.7)	12/72 (16.7)
Above average increase during 2008–2018^e^	8/26 (30.8)	17/32 (53.1)	2/14 (14.3)	27/72 (37.5)
Minimal to average increase during 2008–2018^e^	12/26 (46.2)	7/32 (21.9)	4/14 (28.6)	23/72 (31.9)
Decrease during 2008–2018	4/26 (15.4)	3/32 (9.4)	3/14 (21.4)	10/72 (13.9)
**HEALTH FINANCING**				
Domestic general government health expenditures, per capita in constant 2018 international $ PPP				
High expenditures per capita since 2008^f^	0/25 (0.0)	6/32 (18.8)	12 (80.0)	18/72 (25.0)
Above average growth during 2008–2018^g^	11/25 (44.0)	14/32 (43.8)	2 (13.3)	27/72 (37.5)
Minimal to average growth during 2008–2018^g^	7/25 (28.0)	6/32 (18.8)	0 (0.0)	13/72 (18.1)
Decrease during 2008–2018	7/25 (28.0)	6/32 (18.8)	1 (6.7)	14/72 (19.4)

Acronyms: EML, Essential Medicines List. ILO, International Labour Organization. IQR, interquartile range. MNH, maternal and newborn health. PPP, purchasing power parity. SDG, Sustainable Development Goal.

a Country had all 3 of the following in the EML: oxytocin, misoprostol, and magnesium sulphate.

b Country had all 4 of the following in the EML (or national commodities list): injectable antibiotics, antenatal corticosteroids, chlorhexidine, resuscitation equipment.

c Prior to 2018, the survey asked whether the country has a national policy to notify all maternal deaths. In 2018, the wording changed to ask whether the country has a national policy requiring maternal deaths be notified within 24 h to a central authority. To match historical data, we verified and then recoded 4 entries in the 2018 data to indicate whether the country has any policy on maternal death notification without requiring a specific timeframe within 24 h.

d In 2008, country met SDG index threshold of at least 44.5 doctors, nurses, and midwives per 10,000 population and the value remained at or above the threshold.

e Determination of “above average”, “average”, or “minimal” increase based on 2008–2018 absolute change in density of health personnel.

f In 2008, country government invested at least $150 per capita in health expenditures (top ∼20%) and value remained high or increased.

g Determination of “above average”, “average”, or “minimal” increase based on 2008–2018 rate of growth in GGHE per capita.

**Fig. 2:**
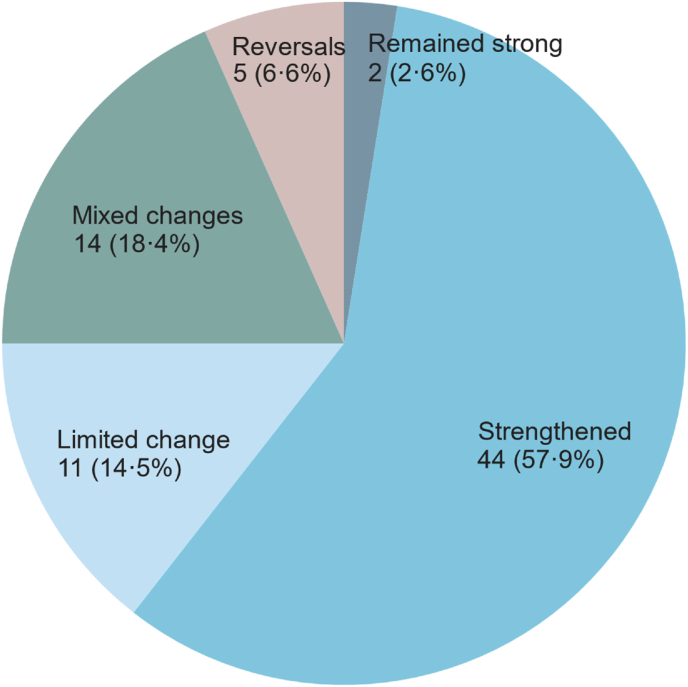
Classification of countries based on overall changes in MNH systems and policy indicators, 2008–2018.

Overall, while more than half of countries (44/76; 57·9%) substantially strengthened the MNH systems and policy environment between 2008 and 2018, some countries (11/76; 14·5%) saw limited change, a few (5/76; 6·6%) saw reversals, and others (14/76; 18·4%) saw both improvements and reversals (Fig. 2). The odds of substantial MNH systems improvement and policy adoption were significantly greater in countries that experienced economic growth between 2008 and 2018 and those that had strong female labor participation and strong governance in 2008 (Table 4). The odds were 1·31 times greater (95% CI: 1·02 to 1·69) for each increase of 10·5% in GDP (i.e., 0·1 ln GDP) per capita. Odds were also significantly greater for countries with higher voice and accountability scores, political stability and absence of violence scores, government effectiveness scores, and rule of law scores in 2008 (all p < 0·05). The share of seats held by women in national parliaments and other country governance indicators (i.e., regulatory quality score, control of corruption score) were not significantly associated with MNH system and policy improvements. The sensitivity analysis found similar results: economic growth and strong country governance showed a significant association with the number of MNH systems and policies that a country had adopted (or attained a minimum threshold) by 2018; female labor participation did not show a significant association in the sensitivity analysis (Supplementary Table S5).

**Table 4:** Odds MNH systems and policy environment strengthened^b^ during 2008–2018, based on economic growth, gender equality, and country governance indicators.

	OR (95% CI)	p-value
**ECONOMIC GROWTH**		
GDP per capita growth, annual % in 2008^a^	1.06 (0.95–1.17)	0.30
Change in ln GDP per capita, PPP, 2008–18 (per 0·1-unit)^c^	1.31 (1.02–1.69)	0.03
**GENDER EQUALITY**		
Labor force participation by females, % in 2008^a^	1.03 (1.00–1.05)	0.03
Seats held by women in national parliaments, % in 2008^a^	1.00 (0.95–1.05)	0.95
Change in % of seats held by women, 2008^a^-18^c^	1.01 (0.95–1.08)	0.62
**COUNTRY GOVERNANCE**		
Voice and accountability score in 2009^a^	2.13 (1.07–4.26)	0.03
Political stability and absence of violence score in 2009^a^	1.80 (1.06–3.06)	0.03
Government effectiveness score in 2009^a^	2.55 (1.01–6.47)	0.05
Regulatory quality score in 2009^a^	1.79 (0.82–3.87)	0.14
Rule of law score in 2009^a^	2.82 (1.10–7.18)	0.03
Control of corruption score in 2009^a^	2.35 (0.87–6.35)	0.09

Acronyms: CI, confidence interval. GDP, gross domestic product. Ln, natural log. MNH, maternal and newborn health. OR, odds ratio. PPP, purchasing power parity.

Notes: Four countries were excluded from the analysis, giving a sample size of 74 countries. Two countries (Brazil and the Maldives) with strong health systems and policy environments that remained strong were excluded, because they are conceptualized to have reached a ceiling wherein further policy adoption and systems strengthening is no longer feasible, irrespective of continued improvement in predictor variables. Two countries (Lesotho and Democratic People's Republic of Korea) were excluded due to lack of data or challenges in validating data.

a 2008 or earliest available date (e.g., South Sudan became an independent country in 2011; 2009 is earliest available year for governance indicators).

b Countries classified as having substantially “strengthened” MNH systems and policies (i.e., at least four of ten indicators adopted/strengthened). The reference group of not strengthened included countries classified as having “limited change”, “mixed changes”, or “reversals”.

c For economic growth, change was measured as the difference in ln GDP per capita between 2008 and 2018, rescaled to measure changes per 0·1-units of ln GDP per capita; this is equivalent to a change of 10.5% in GDP per capita. For gender equality, change was measured as the difference in absolute percentages between 2008 and 2018.

## 4 Discussion

In the decade following 2008, many LMICs adopted policies and strengthened systems relevant for MNH. More than half substantially strengthened policies and systems, as signaled by the adoption of four or more of the ten tracer indicators analyzed in this study. Changes were most notably seen in the widespread adoption of national guidelines for kangaroo mother care and use of antenatal corticosteroids, widespread adoption of national policies for maternal death notification and review, and the introduction of priority MNH medicines in EMLs. Few LMICs had the aforementioned policies in place in 2008, but most had adopted these by 2018. This timeframe corresponds with the launch of important global strategies and initiatives. In 2010, former UN Secretary-General Ban Ki-moon launched the *Every Woman Every Child* (EWEC) movement to mobilize action for women's, children's, and adolescents' health. This was followed by the establishment of the UN Commission on Life-Saving Commodities for Women and Children and the UN Commission on Information and Accountability, the launch of ENAP and EPMM, and the publication of various technical guidance documents and recommendations (StataCorp, 2017; World Health Organization, 2015). The six aforementioned indicators align closely with priority actions and indicators identified and championed by this movement.

Progress has been slower on maternity leave legislation and regulations on the marketing of breast-milk substitutes. The ILO adopted Convention No. 183 (C183) on maternity protection in 2000, and maternity benefit coverage is also now an indicator for the SDGs (1.3.1). While most countries have enacted at least some legal provisions for maternity leave, the duration and level of benefits are often limited and eligibility restricted to those employed in the formal economy. Coverage is worse in low-income countries, where only 10·5% of women giving birth received maternity care benefits in 2020 (International Labour Organization, 2021). Forty years after its endorsement by the World Health Assembly in 1981, progress has likewise been slow in fully enacting the International Code of Marketing of Breast-milk Substitutes. The slower progress for these two indicators could reflect the difficulty of passing legislation—which often involves navigating complex political and fiscal obstacles. Corporate influences can also be an obstacle to enacting legislation regulating labor and marketing practices (World Health Organization, 2013b; Pereira-Kotze et al., 2022; Baker et al., 2021a; Baker et al., 2021b).

Delivering maternal and newborn health services depends on having adequate financial and human resources for health. A systemic bottleneck analysis conducted with over 600 experts as part of the Lancet's Every Newborn Series identified health workforce and financing as common constraints to MNH care (Baker et al., 2021c). Our results showed an overall increase in health workforce density and government health spending per capita, but there was substantial variation across countries. Some countries saw rapid growth while others experienced reversals. Beyond those countries that had already attained the SDG threshold of at least 44·5 physicians, nurses, and midwives per 10,000 population in 2008 (Dickson et al., 2014), no additional countries achieved the SDG threshold for health workforce density by 2018.

Countries that substantially strengthened the MNH systems and policy environment between 2008 and 2018 were more likely to be those that also experienced economic growth and had strong country governance. An analysis of data from 143 countries over 14 years found that GDP growth was associated with increased government health expenditures (World Health Organization, 2016b). Furthermore, previous studies have found that country governance is associated with improvements in the coverage of health interventions (Ke et al., 2011), and mortality reductions (Kuruvilla et al., 2014; Bishai et al., 2016; Andrus et al., 2020). Together, this research supports the theory that good country governance and economic growth create an environment conducive to the adoption of new policies, health workforce investments, and systems improvements important for MNH.

However, the mere adoption of MNH-friendly policies and strengthened systems are not sufficient to improve MNH outcomes. While enacting favorable policies and improving systems can create an enabling environment that facilitates the scale-up of effective interventions, it does not guarantee robust implementation nor widespread coverage of these interventions. For example, Torres et al. described achievements integrating kangaroo mother care into national policy in six priority countries, but noted the proportion of small newborns initiated in kangaroo mother care remains low (Ruiz-Cantero et al., 2019). Studies have also documented violations of legal regulations against the marketing of breastmilk substitutes across many countries, highlighting the need for stronger monitoring and enforcement of existing regulations (Baker et al., 2021b; Torres et al., 2021; Changing Markets Foundation, 2017; International Baby Food Action Network IBFAN, 2017; Pries et al., 2016)(Champeny et al., 2016).

Our study provides a useful point of entry for understanding the MNH policy and systems environment globally, but country-specific analyses are likewise critical to understanding the political, social, economic, and health system determinants that influence MNH within the local context. Our study analyzes data from several sources, including a global policy survey of WHO Member States on laws, policies, and strategies relevant for MNH. This data is also available to the public on the Maternal, Newborn, Child, and Adolescent Health and Ageing Data Portal (https://platform.who.int/data/maternal-newborn-child-adolescent-ageing/national-policies). The portal allows users to review country-specific health systems and policy indicators, visualize data using charts and maps, and search a repository of national laws, strategies, and policy documents. Such resources can provide policymakers, implementers, and researchers with information on the legal and policy environment in different countries, and they can facilitate future analyses and dialogue on policy options to improve MNH.

Analyzing a country's health system and policy environment is complex. The multitude of diverse laws, regulations, policies, and systems that can influence MNH in any given country are numerous. This study is limited to assessing ten MNH policy and systems indicators, which necessarily omits important policy and system variables that may have greater impact on MNH than those studied. Furthermore, there are many intangible and context-specific variables critical for understanding why country governments adopt policies or invest in health systems, and what makes these policies and systems effective (or not). This study is limited by its focus on a few, tangible variables that can be measured and quantified.

Another limitation of the study is the variability of data quality. Many indicators are self-reported by country teams; often, representatives from the Ministry of Health and/or focal points at various partners worked together to complete policy surveys and respond to requests for information (Katwan et al., 2021). Validation exercises were conducted to verify self-reported responses against country laws, policies, and guidelines. This included a detailed validation of responses for selected survey questions in the 2018 SRMNCAH policy survey, as described by Katwan and colleagues (Katwan et al., 2021). An additional validation exercise was conducted for questions relevant to this study across previous policy survey rounds, but it was not possible to verify self-reported data for all responses across all years. In addition, the types of questions included in the WHO SRMNCAH policy surveys and their phrasing has changed over the years. Relevant country laws, policies, and guidelines were reviewed to complete or verify information that was missing for certain years or was reported inconsistently across surveys.

## 5 Conclusions

Overall, our study finds the policy and systems landscape for MNH is changing in many LMICs. We document widespread adoption of several priority MNH policies and guidelines, including guidelines for kangaroo mother care and antenatal corticosteroids, policies related to maternal death surveillance and response systems, and the integration of priority maternal and newborn medicines on EMLs. The adoption of these policies and guidelines are a notable step in creating an environment supportive of MNH, but continued leadership and resources are needed to strengthen implementation of these policies and guidelines. Dialogue to understand and address the barriers and facilitators encountered by actors at different levels of the health system can strengthen implementation and help ensure that evidence-based policies and guidelines translate into tangible improvements for maternal and newborn health and wellbeing.

## Author contributions

Elizabeth Stierman: Conceptualization, Methodology, Formal analysis, Writing – original draft. Blerta Maliqi: Conceptualization, Methodology, Writing – review & editing. Meighan Mary: Conceptualization, Methodology, Writing – review & editing. Martin Dohlsten: Conceptualization, Methodology, Writing – review & editing. Elizabeth Katwan: Conceptualization, Methodology, Data curation, Writing – review & editing. Allisyn Moran: Conceptualization, Methodology, Writing – review & editing, Supervision. Andreea Creanga: Conceptualization, Methodology, Writing – review & editing, Supervision.

## Funding

This work was supported, in whole or in part, by the 10.13039/100000865Bill & Melinda Gates Foundation [INV-009058].

## Disclaimer

The authors alone are responsible for the views expressed in this article and they do not necessarily represent the views, decisions or policies of the institutions with which they are affiliated.

## Declaration of competing interest

The authors declare that they have no known competing financial interests or personal relationships that could have appeared to influence the work reported in this paper.

## Data Availability

Data will be made available on request.
