# Supplementary material for: Changes in the health systems and policy environment for maternal and newborn health, 2008–2018: An analysis of data from 78 low-income and middle-income countries
Source: Soc Sci Med. 2023 Mar;321:115765. doi: 10.1016/j.socscimed.2023.115765 (PMC10024243; doi:10.1016/j.socscimed.2023.115765)
Supplement: Multimedia component 1 [file mmc1.docx]

**SUPPLEMENTARY MATERIAL**

**TABLE OF CONTENTS**

[Table S1. Indicator definitions and data sources 2](#_Toc109722539)

[Table S2. Classification scheme for rating overall change in MNH systems and policy environment 4](#_Toc109722540)

[Table S3. Economic growth, gender equality, and country governance indicators, 2008-2018 5](#_Toc109722541)

[Table S4. MNH systems and policy indicators, 2008-2018 6](#_Toc109722542)

[Table S5. Association between the MNH systems and policies environment in 2018† and indicators of economic growth, gender equality, and country governance during the preceding decade (2008-18) 8](#_Toc109722543)

# **Table S1. Indicator definitions and data sources**

| Categories | Indicators | Measurement | Data sources | Time points |
| --- | --- | --- | --- | --- |
| **MNH SYSTEMS AND POLICY INDICATORS** | | | | |
| Legislative context | Paid maternity leave legislation in alignment with ILO Convention 183 (C183) | Categorical variable indicating the level of alignment with international convention: (a) ratified ILO C183; (b) substantially aligned^1^; (c) moderately aligned^2^; (d) few or no provisions in law^3^ | ILO World Social Protection Reports & ILO Maternity and Paternity at Work Reports (provides 2013-2018 data); ILO Working Conditions Laws Database (provides 2008-2012 data); ILO NORMLEX database (provides data on ratifications) | Annually 2008-2018 |
|  | Legal status of International Code of Marketing of Breastmilk Substitutes | Categorical variable indicating the level of alignment with the Code^4^: (a) substantially aligned with the Code; (b) moderately aligned with the Code; (c) some provisions of the Code adopted; (d) no provisions in law | WHO & UNICEF Marketing of Breast‑milk Substitutes: National Implementation of the International Code Status Reports | Annually 2008-2018 |
| MNH governance and leadership | National guidelines recommend Kangaroo Mother Care for clinically stable low birthweight newborns | Binary variable indicating the existence (or not) of the guidelines | WHO SRMNCAH policy surveys (provides 2014/-2018 data); Healthy Newborn Network (provides data prior to 2014) | 2008, 2015, 2018 |
|  | National guidelines recommend use of antenatal corticosteroids for preterm labor | Binary variable indicating the existence (or not) of the guidelines | WHO SRMNCAH policy surveys (provides 2014-2018 data); Healthy Newborn Network (provides data prior to 2014) | 2008, 2015, 2018 |
| MNH medicines and commodities | Priority maternal medicines included in the Essential Medicine List | Binary variable indicating the inclusion (or not) of all 3 priority medicines—oxytocin, misoprostol, and magnesium sulphate—in the Essential Medicine List | WHO SRMNCAH policy surveys | 2015, 2018 |
|  | Priority newborn medicines included in the Essential Medicine List | Binary variable indicating the inclusion (or not) of all 4 priority medicines and commodities— injectable antibiotics, antenatal corticosteroids, chlorhexidine, resuscitation equipment—in the Essential Medicine List (or National Commodities List) | WHO SRMNCAH policy surveys | 2015, 2018 |
| Maternal death surveillance and response | National policy requires notification of maternal deaths | Binary variable indicating the existence (or not) of the policy^5^ | WHO SRMNCAH policy survey (provides 2018-2019 data); WHO Global MDSR implementation survey (provides data for 2015 and prior years) | Annually 2008-2015, and 2018 |
|  | National policy requires review of maternal deaths | Binary variable indicating the existence (or not) of the policy | WHO SRMNCAH policy survey (provides 2018-2019 data); WHO Global MDSR implementation survey (provides data for 2015 and prior years) | Annually 2008-2015, and 2018 |
| Health workforce | Density of physicians, nurses, and midwives per 10,000 population | Continuous variable indicating number of physicians, nurses, and midwives per 10,000 population | WHO Global Health Workforce Statistics database | Annually 2008-2018 |
| Health financing | Domestic general government health expenditures per capita, PPP (constant 2018 international $) | Continuous variable indicating domestic general government health expenditures per capita based on purchasing power parity (PPP) | WHO Global Health Expenditure Database (GHED) | Annually 2008-2018 |
| CONTEXTUAL FACTORS | | | | |
| Economics | Gross domestic product (GDP) per capita, PPP (constant 2017 international $) | Continuous variable indicating GDP per capita based on PPP | World Bank’s World Development Indicators database | Annually 2008-2018 |
|  | GDP per capita growth, annual % | Continuous variable indicating annual percentage growth rate of GDP per capita based on constant local currency | World Bank’s World Development Indicators database | Annually 2008-2018 |
| Gender equality | Seats held by women in national parliaments (%) | Percentage of parliamentary seats in a single or lower chamber held by women | World Bank’s World Development Indicators database [original source: Inter-Parliamentary Union (IPU)] | Annually 2008-2018 |
|  | Labor force participation by females (%) | Percentage of the female population ages 15 and older that is economically active | World Bank’s World Development Indicators database [original source: International Labour Organization, ILOSTAT database] | Annually 2008-2018 |
| Governance (macro-level; not specific to health sector) | - Voice and accountability score - Political stability and absence of violence score - Government effectiveness score - Regulatory quality score - Rule of law score - Control of corruption score | Each score is a composite measure constructed by weighting and averaging data from underlying sources related to the given concept of governance; scores are measured in units of a standard normal distribution (mean zero) | Worldwide Governance Indicators project | 2009, 2014, 2019 |

^1^ Country has passed national legislation that is aligned with three key provisions of ILO convention 183 (14 weeks of maternity leave, paid at 66% of previous earnings or higher, fully financed by social insurance or public funds).

^2^ Country has ratified the earlier ILO Convention 103 or has passed national legislation that is aligned with three key provisions of the convention (12 weeks of maternity leave, paid at 66% of previous earnings or higher, at least partially financed social insurance or public funds).

^3^ Country had not passed national legislation on maternity leave, or country has passed legislation that only weakly aligns with ILO conventions (e.g., program is employer liability).

^4^ Classification of legislation harmonized with definitions used in the WHO & UNICEF International Code Status Report 2020; previous years were updated to match 2020 definitions.

^5^ Prior to 2018, the survey asked whether the country has a national policy to notify all maternal deaths. In 2018, the wording changed to ask whether the country has a national policy requiring maternal deaths be notified within 24 hours to a central authority. To match historical data, we verified and then recoded 4 entries in the 2018 data to indicate whether the country has any policy on maternal death notification without requiring a specific timeframe within 24 hours.

# **Table S2. Classification scheme for rating overall change in MNH systems and policy environment**

| Short Name | Long Name | Definition |
| --- | --- | --- |
| Remained strong | Strong MNH systems and policy environment that remained strong | Country meets both criteria:   - In 2008^1^, at least 6 of the 10 tracer indicators were already in existence or had attained the target threshold. - No reversals were reported in any of the 10 indicators. |
| Strengthened | MNH systems and policy environment that became substantially stronger† | Option A - country meets both criteria:   - During 2008-2018, improvements^2^ were seen in at least 4 tracer indicators. - No reversals were reported in any of the 10 indicators.   Option B - country meets both criteria:   - During 2008-2018, improvements^2^ were seen in at least 5 tracer indicators. - One reversal reported, but small in size. |
| Limited change | MNH systems and policy environment with minimal or no change | Option A - country meets both criteria:   - During 2008-2018, improvements^2^ were seen in 3 or fewer tracer indicators. - No reversals were reported in any of the 10 indicators.   Option B - country meets both criteria:   - During 2008-2018, improvements^2^ were seen in 4 or fewer tracer indicators. - One reversal reported, but small in size. |
| Reversals | MNH systems and policy environment with substantial reversals | Country meets both criteria:   - Minimal or no improvement in tracer indicators. - At least one large reversal. |
| Mixed changes | MNH systems and policy environment with mixed changes‡ | Country meets both criteria:   - During 2008-2018, improvements^2^ were seen in at least 4 tracer indicators. - At least one large reversal reported. |

^1^ 2008 or earliest available date.

^2^ Improvements include strengthening MNH legislation, adoption of MNH policies or guidelines, addition of priority MNH medicines in Essential Medicines Lists, higher-than-average increase in health workforce density, and higher-than-average growth in domestic general government health expenditures.

# **Table S3. Economic growth, gender equality, and country governance indicators, 2008-2018**

|  | 2008/2009† | 2014 | 2018/19‡ |
| --- | --- | --- | --- |
| **ECONOMIC GROWTH** | **Median (IQR)** | **Median (IQR)** | **Median (IQR)** |
| GDP per capita growth, annual % | 4·0 (1·5-6·4) | 2·6 (1·0-4·4) | 2·3 (0·4-4·1) |
| GDP per capita, PPP (2017 constant international $) | 3,470 (1,899-7,413) | 4,167 (2,194-9,688) | 4,884 (2,543-11,004) |
| **GENDER EQUALITY** | **Mean (SD)** | **Mean (SD)** | **Mean (SD)** |
| Seats held by women in national parliaments, % | 16·7 (10·1) | 20·0 (11.9) | 21·2 (12·0) |
| Labor force participation by females, % females ages 15+ | 54·5 (19·6) | 54·0 (19.0) | 54·0 (19·3) |
| **COUNTRY GOVERNANCE^1^** | **Mean (SD)** | **Mean (SD)** | **Mean (SD)** |
| Voice and accountability score | -0·75 (0·75) | -0·71 (0·76) | -0·70 (0·77) |
| Political stability and absence of violence score | -0·69 (0·93) | -0·73 (0·87) | -0·71 (0·86) |
| Government effectiveness score | -0·77 (0·54) | -0·78 (0·59) | -0·78 (0·64) |
| Regulatory quality score | -0·73 (0·64) | -0·72 (0·60) | -0·74 (0·61) |
| Rule of law score | -0·81 (0·56) | -0·75 (0·53) | -0·79 (0·58) |
| Control of corruption score | -0·76 (0·53) | -0·77 (0·57) | -0·78 (0·60) |

Acronyms: IQR, interquartile range. PPP, purchasing power parity. SD, standard deviation.

† 2008 values are shown for all indicators except governance indicators which reflect 2009 value.

‡ 2018 values are shown for all indicators except governance indicators which reflect 2019 value.

^1^ Governance indicators are reported in their standard normal units, ranging from approximately -2·5 to 2·5. Note the standard normal distribution is based on data from all countries in the Worldwide Governance Indicators database; therefore, the distribution of scores for the 78 LMICs studied in this paper may not have a mean of 0 or SD of 1.

# **Table S4. MNH systems and policy indicators, 2008-2018**

|  | **2008** | **2010** | **2012** | **2014/15†** | **2016** | **2018** |
| --- | --- | --- | --- | --- | --- | --- |
|  | **n=77** | **n=77** | **n=78** | **n=78** | **n=78** | **n=78** |
| **LEGISLATIVE CONTEXT** | **n (%)** | **n (%)** | **n (%)** | **n (%)** | **n (%)** | **n (%)** |
| Paid maternity leave legislation in alignment with ILO Convention 183 (C183) |  |  |  |  |  |  |
| Ratified ILO C183 | 2/76 (2·6) | 3/76 (4·0) | 5/77 (6·5) | 6/77 (7·8) | 7/77 (9·1) | 8/77 (10·4) |
| Substantially aligned^1^ | 15/76 (19·7) | 14/76 (18·4) | 14/77 (18·2) | 14/77 (18·2) | 17/77 (22·1) | 17/77 (22·1) |
| Moderately aligned^2^ | 22/76 (29·0) | 22/76 (29·0) | 20/77 (26·0) | 19/77 (24·7) | 21/77 (27·3) | 21/77 (27·3) |
| Few or no provisions in law^3^ | 37/76 (48·7) | 37/76 (48·7) | 38/77 (49·3) | 38/77 (49·3) | 32/77 (41·6) | 31/77 (40·3) |
| Legal status of International Code of Marketing of Breast-milk Substitutes^4^ |  |  |  |  |  |  |
| Substantially aligned with the Code | 11 (14·3) | 12 (15·6) | 14 (18·0) | 16 (20·5) | 16 (20·5) | 17 (21·8) |
| Moderately aligned with the Code | 18 (23·4) | 19 (24·7) | 22 (28·2) | 25 (32·1) | 25 (32·1) | 25 (32·1) |
| Some provisions of Code adopted | 15 (19·5) | 16 (20·8) | 16 (20·5) | 15 (19·2) | 16 (20·5) | 15 (19·2) |
| No provisions in law | 33 (42·9) | 30 (39·0) | 26 (33·3) | 22 (28·2) | 21 (26·9) | 21 (26·9) |
| **MNH GOVERNANCE AND LEADERSHIP** |  |  |  |  |  |  |
| National guidelines recommend Kangaroo Mother Care for clinically stable low birthweight newborns | 2 (2·6) | NA | NA | 40/65 (61·5) | NA | 64/76 (84·2) |
| National guidelines recommend use of antenatal corticosteroids for preterm labor | 1 (1·3) | NA | NA | 39/55 (70·9) | NA | 69/76 (90·8) |
| **MNH MEDICINES AND COMMODITIES** |  |  |  |  |  |  |
| Priority maternal medicines included in the EML^5^ | NA | NA | NA | 42/69 (60·9) | NA | 73 (93·6) |
| Priority newborn medicines included in the EML^6^ | NA | NA | NA | 5/68 (7·4) | NA | 57/72 (79·2) |
| **MATERNAL DEATH SURVEILLANCE AND RESPONSE** |  |  |  |  |  |  |
| National policy to notify all maternal deaths^7^ | 27/68 (39·7) | 42/74 (56·8) | 48/76 (63·2) | 69 (88·5) | NA | 75 (96·2) |
| National policy to review maternal deaths | 17/52 (32·7) | 26/52 (50·0) | 33/52 (63·5) | 49/55 (89·1) | NA | 76 (97·4) |
| **HEALTH WORKFORCE** | **Median (IQR)** | **Median (IQR)** | **Median (IQR)** | **Median (IQR)** | **Median (IQR)** | **Median (IQR)** |
| Aggregate density of physicians, nurses and midwives per 10,000 population^8^ | 11·9 (6·1-29·1) | 13·3 (6·7-29·3) | 14·4 (7·2-29·6) | 15·1 (7·7-30·8) | 15·8 (8·3-32·5) | 17·0 (9·1-32·3) |
| **HEALTH FINANCING** |  |  |  |  |  |  |
| Domestic general government health expenditures, per capita in PPP international dollars^8^ | 41·3 (21·5- 155·6) | 47·8 (20·9-168·8) | 54·2 (22·9-179·4) | 60·7 (24·0-189·5) | 66·8 (25·0-202·5) | 71·2 (27·8-214·7) |

Acronyms: EML, Essential Medicines List. ILO, International Labour Organization. IQR, interquartile range. NA, not applicable. PPP, purchasing power parity.

Notes: Sample includes 77 countries prior to 2011, and 78 countries in 2011 or later years; South Sudan became an independent country in 2011.

† 2014 values are shown for all indicators except national guidelines (kangaroo mother care, antenatal corticosteroids), priority medicines (maternal, newborn), and national policies (maternal death notification, maternal death review), which reflect 2015 value.

^1^ Country has passed national legislation that is aligned with three key provisions of ILO convention 183 (14 weeks of maternity leave, paid at 66% of previous earnings or higher, fully financed by social insurance or public funds).

^2^ Country has ratified the earlier ILO Convention 103 or has passed national legislation that is aligned with three key provisions of the convention (12 weeks of maternity leave, paid at 66% of previous earnings or higher, at least partially financed social insurance or public funds).

^3^ Country had not passed national legislation on maternity leave, or country has passed legislation that only weakly aligns with ILO conventions (e.g., program is employer liability).

^4^ Classification of legislation harmonized with definitions used in the WHO & UNICEF International Code Status Report 2020; previous years were updated to match 2020 definitions.

^5^ Country has all 3 of the following in the Essential Medicines List: oxytocin, misoprostol, and magnesium sulphate.

^6^ Country has all 4 of the following in the Essential Medicines List (or National Commodities List): injectable antibiotics, antenatal corticosteroids, chlorhexidine, resuscitation equipment.

^7^ Prior to 2018, the survey asked whether the country has a national policy to notify all maternal deaths. In 2018, the wording changed to ask whether the country has a national policy requiring maternal deaths be notified within 24 hours to a central authority. To match historical data, we verified and then recoded 4 entries in the 2018 data to indicate whether the country has any policy on maternal death notification without requiring a specific timeframe within 24 hours.

^8^ Annual country estimates predicted using a mixed-effects model with a random intercept for country and random slope for time.

# **Table S5. Association between the MNH systems and policies environment in 2018† and indicators of economic growth, gender equality, and country governance during the preceding decade (2008-18)**

|  | Coefficient (95% CI) | p-value |
| --- | --- | --- |
| **ECONOMIC GROWTH** |  |  |
| Change in ln GDP per capita, PPP, 2008-18 (per 0.1-unit)‡ | 0·12 (0·00 to 0·24) | 0·04 |
| **GENDER EQUALITY** |  |  |
| Labor force participation by females, % | 0·00 (-0·02 to 0·01) | 0·74 |
| Seats held by women in national parliaments, % | 0·01 (-0·02 to 0·04) | 0·45 |
| **COUNTRY GOVERNANCE** |  |  |
| Voice and accountability score | 0·58 (0·17 to 0·98) | <0·01 |
| Political stability and absence of violence score | 0·57 (0·22 to 0·92) | <0·01 |
| Government effectiveness score | 1·17 (0·69 to 1·65) | <0·001 |
| Regulatory quality score | 1·01 (0·53 to 1·48) | <0·001 |
| Rule of law score | 0·84 (0·28 to 1·39) | <0·01 |
| Control of corruption score | 0·53 (-0·03 to 1·10) | 0·06 |

Acronyms: CI, confidence interval. GDP, gross domestic product. Ln, natural log. MNH, maternal and newborn health. PPP, purchasing power parity.

Notes: Indicators (predictor variables) are measured using their average value over the reference period: 2008-18 for economic growth and gender equality indicators; 2009-19 for country governance indicators.

† Outcome measures the extent that MNH systems and policies are available—or attained threshold values—by the end of the reference period in 2018. The score is calculated using 10 tracer MNH systems and policy indicators, with a minimum value of 0 and maximum of 10. Two indicators are defined by threshold values: (1) aggregate density of physicians, nurses and midwives per 10,000 population (1 point given for reaching SDG index threshold of at least 44·5 per capita); and (2) domestic general government health expenditures (1 point given for reaching threshold of $150 per capita). The other eight indicators are given a 1 for having the policy in 2018, or 0 for not having the policy in 2018; partial scores are given for legislation that is partially aligned/adopted.

‡ For economic growth, change was measured as the difference in ln GDP per capita between 2008 and 2018, rescaled to measure changes per 0·1-units of ln GDP per capita; this is equivalent to a change of 10·5% in GDP per capita. For gender equality, change was measured as the difference in absolute percentages between 2008 and 2018.
